# Location-specific pathology analysis of monopodial airways in a rabbit model of bronchopulmonary dysplasia: a proof of principle study

**DOI:** 10.1186/s12880-025-01657-6

**Published:** 2025-04-14

**Authors:** Yannis Pfleger, Lena S. C. Bode, David Haberthür, Ruslan Hlushchuk, Yannick Regin, Andre G. Gie, Thomas Salaets, Jaan Toelen, Christian Mühlfeld, Jonas Labode

**Affiliations:** 1 Institute of Functional and Applied Anatomy, Hannover Medical School, Carl-Neuberg-Str. 1, 30625 Hannover, Germany; 2 Institute of Anatomy, University of Bern, Baltzerstrasse 2, Bern, 3012 Switzerland; 3 Department of Development and Regeneration, KU Leuven, Leuven, 3000 Belgium; 4 Department of Paediatrics and Child Health, Faculty of Medicine, Stellenbosch University, Cape Town, South Africa; 5 Member of the German Center for Lung Research (DZL), Biomedical Research in Endstage and Obstructive Lung Disease (BREATH), Hannover, Germany

**Keywords:** Airways, Monopodial lung, Cluster analysis, Branching analysis, Micro computed tomography, Light microscopy, Bronchopulmonary dysplasia

## Abstract

**Background:**

The airways of the mammalian lung form a tree-like structure, starting from the trachea and branching out to the terminal bronchioles. This tree is composed of heterogeneous sub-structures or compartments, varying in morphological characteristics such as composition of airway epithelium, presence of cartilage plates, and number of smooth muscle cell layers or lumen diameter. These compartments may vary in their reaction to different pathological stimuli. Thus, when studying a particular lung disease, the compartments need to be investigated individually and not as part of a more global portmanteau compartment. In the symmetrically branching primate lungs, dividing the airway tree into generations is a common method to create morphologically homogeneous groups of airway segments. In common lab animals however, an asymmetrical branching pattern is present, where conventional branching-based grouping methods are unable to create meaningful results.

**Methods:**

Therefore, a morphological clustering approach was tested in the current proof of principle study for its suitability of dividing airways into biologically meaningful sub-compartments. On this basis, an investigation of the distribution of pulmonary airway changes in a bronchopulmonary dysplasia rabbit model was conducted.

**Results:**

The approach of clustering airway segments by morphology instead of branching pattern proved to be capable of creating meaningful airway compartments. This way, the distribution of differences that would not have been visible in a purely global comparison of morphological characteristics, could be identified between disease model and control group.

**Conclusions:**

The employed clustering model is applicable to study the contribution of airway sub-compartments in pulmonary diseases. On this basis, targeted strategies for their mitigation may be developed.

## Background

The mammalian lung is composed of morphologically different sub-structures [1]. Conductive and respiratory compartments can be distinguished. But even within purely conductive airways, there is no uniform morphological composition. The epithelium changes in size and cell population over the course through the organ, as do e.g. cartilage content, smooth muscle cell number, and the overall diameter of the airway [2]. It is thus not surprising that different airway segments are susceptible to diseases in different ways. They manifest in the range from bronchomalacia and atelectasis in the mainstem bronchi down to mucus plugging and manifestations of sarcoidosis in the bronchioles. Further examples include localized inflammation (e.g. bronchiolitis), hyperinflation or meta-, hypo- and hyperplasia of specific cell populations [3–6]. Therefore, in studies on lung diseases, comparisons of airway segments with equal function and morphology could provide a more detailed and better understanding of the underlying disease processes.

In the relatively symmetrically branching airways of the human lung, the aforementioned functional units can be roughly approximated by identifying generations of airways and grouping them accordingly. Literature gives estimates for the major morphological transitions within the airways [7]. The first three generations are bronchi, generations 4 to 14 are bronchioles, generation 15 marks the last section of the conductive airways with the terminal bronchioles. Generations 16 to 18 contain the respiratory bronchioles and so forth. However, it should be pointed out that other authors indicate different generations for these transitions [8].

Horsfield and Cumming [9] criticized the generational approach, as it assumes a regular dichotomy and thus excludes any asymmetry that might be present in the branching pattern. This in turn would lead to grouping of differently sized branches in the same generation. They demonstrated the extent of this asymmetry in the human lung by determining the number of divisions of the airway on its path down to a specified branch diameter of 7 mm. While the mode of the resulting distribution amounted to 14 divisions, observations ranged from 8 to 25 divisions. This highlights the importance of incorporating asymmetrical branching patterns into models used in lung research.

The situation is even more extreme outside of humans and other primates. The lungs of common laboratory animals, such as e.g. mice and rabbits, exhibit a highly asymmetric (monopodial) branching pattern, characterized by one long central airway per lobe with lateral daughter branches [10, 11].

Just like for the human lung [12], a nomenclature for the broncho-pulmonary airways exists for these animals. On this basis, individual segment comparisons are possible [13], but might be hindered by pattern variations [14]. While useful for the comparison of individual major airway branches, for the task of defining homogeneous sub-compartments, it is comparable to the generational approach.

In a previous study, Labode et al. [15] demonstrated, that common grouping methods such as generations [16] and orders [17] perform poorly at the task of separating the monopodial pulmonary artery into homogeneous subgroups. While methods specifically aimed at asymmetrical branching patterns, such as fractal generations [18] and Strahler orders [19] performed better, their results were still not satisfactory. On the other hand, the use of a clustering algorithm proved promising. Thus, this approach was employed to compare the pulmonary vasculature of an experimental and a control group in a pilot study for a rabbit model of bronchopulmonary dysplasia (BDP) [20]. The method proved to produce meaningful results, delivering insights into differences in vascular morphology between the two groups. Compared to an unfocused, global analysis approach, this method was able to deliver a higher precision and in some cases revealed local differences between groups, that were undetectable in the global analysis.

As airways and arteries mostly branch in parallel [21], it was expected, that the method pioneered on the vasculature would produce meaningful results in the airways as well. Thus, to further evaluate the capabilities of this method, it was applied to the conducting airways of the experimental rabbit dataset in the current proof of principle study. The goal was to evaluate its performance on this target. It was to be tested whether the clustering approach is able to divide the airway trees into meaningful and comparable morphological sub-areas. If so, then the group-based comparison could deliver results unobtainable with a global analysis. Only then, the method can be judged as useful and justify the resulting increased workload.

The experimental context, in which this study is embedded, is a BPD research project.

A model incorporating premature birth and hyperoxia has been established in recent years and is able to approximate BPD in the rabbit and study disease onset and progression [22, 23]. The rabbit constitutes the smallest animal model of BPD that incorporates prematurity, allowing both explorative and translational research [24]. Changes in the rabbit lung that are known to occur due to this treatment affect e.g. airway morphology [25], epithelium, or smooth muscle cells [26]. Thus, it is deemed an appropriate test case for the method.

The target of this study are the conducting airways, namely the structures of the main bronchus down to the terminal bronchioles. These are comparable in their gross morphology to the vasculature studied previously and are compatible with the computational tools developed in this context. They consist of tube-like segments with a tree-like branching structure and dimensions that can be imaged using micro computed tomography (*µ*CT). As the available image resolution and computational tools of this study prohibit the analysis of the gas exchange regions, they will therefore be excluded. This separation of conductive airways from gas exchange regions is a well established process in morphometric lung studies. In the past it has often been performed by physically cutting away respiratory sections from corrosion casts of the lung [11].

This study is preclinical in nature and aims at laying the methodical groundwork for better uncovering the pathophysiology of BPD. Nevertheless, computed tomography (CT) scans of the lung in general [27, 28] and CT scans of the airways in specific [29], have been used in a diagnostic role in clinical settings and have been able to assess disease severity in infants with chronic lung disease. The methods employed here for airway comparison may serve as an extension to these diagnostic tools.

## Methods

### Rabbit BPD model

The organs used for this study stem from a rabbit BPD model. The experiment was approved by the Ethics committee for Animal Experimentation of KU Leuven, Belgium (P081/2017). The rabbits were purchased from the KU Leuven animal facility.

An extensive description of the rabbit treatment and lung sample generation is available in [30]. Major elements were the delivery of n = 4 rabbit pups by cesarean section on day 28 of pregnancy (term = 31 days). They were placed in an incubator (Okolab, Italy) with controlled environmental conditions of 32 °C and 50% humidity. One group (n = 2) was kept in a normoxic (NOX) atmosphere (21% O_2_), while the other group was exposed to a hyperoxic (HYX) atmosphere (>95% O_2_). On day seven, the animals were anesthetized with intramuscular ketamine (35 mg/kg) and xylazin (6 mg/kg). Afterwards, perfusion fixation of the lungs with an aldehyde fixative at a pressure of 25 cm H_2_O was performed. Death occurred during this process by exsanguination. The heart-lung block was then removed and divided. The left lungs were used for the current study, the right lungs were prepared for another, electron microscopic, study.

For the purpose of the current study, the left lungs were post-fixed using 1% osmium tetroxide and 1% uranyl acetate. After dehydration in an ascending acetone series, the lungs were individually embedded in glycol methacrylate (Technovit 7100, Heraeus Kulzer, Wehrheim, Germany).

### *µ***CT image acquisition**

After embedding, the lungs were imaged non-destructively using *µ*CT. Scans were performed on a SkyScan 1272 high-resolution microtomograph (Control software version 1.1.19, Bruker microCT, Kontich, Belgium). Reconstruction of the acquired projection images was conducted using NRecon (Version 1.7.4.2, Bruker microCT, Kontich, Belgium). The isometric voxel size of the resulting datasets was 7 µm.

### Lung sectioning and light microscopy

To produce material for morphological feature acquisition, a randomized sampling scheme was employed [31]. The lungs were cut on a microtome parallel to the longitudinal axis. Sample series of 24 slices each were collected with a thickness of 4 *µ*m for two animals (labels NOX 1 and HYX 1) and 2 µm for the remaining two (NOX 2 and HYX 2). Starting from a random point between 0 *µ*m and the inter-series distance of 200 *µ*m for NOX 1 and HYX 1 or 100 *µ*m for NOX 2 and HYX 2, a sample series was collected. The inter-series distance was then discarded and the next sample series was collected. This process was repeated until the whole lung had been sectioned. The different distances and slice thicknesses were defined as there was no previous experience with the tissue registration and morphological acquisition performed in this study. The collected sample tissue slices were mounted on glass slides and stained using toluidine blue.

Slides prepared in this fashion were then scanned on an AxioScan.Z1 slide scanner (Zeiss, Germany) to generate digital light microscopy (LM) images. All slides were scanned once at 20x objective lens magnification for registration purposes. The center slides of each 24 slice stack were additionally scanned at 40x objective lens magnification for an airway epithelium analysis.

### Image segmentation and isolation of the conducting airways

The *µ*CT image sets were processed as described by Grothausmann et al. [30]. In short, they were pre-segmented using an Insight Toolkit (ITK) [32] watershed filter. All resulting watershed labels within the airways were then joined to one continuous airway label using ITK-SNAP v3.4 [33] with the Click’n’Join mode plugin [34]. The resulting 3D model consisted of the luminal airway space, starting from the main bronchus and extending all the way into the acini. There, the thin alveolar walls were not sufficiently resolved anymore for a correct segmentation. This was caused by the available *µ*CT resolution in combination with the achievable contrast and noise levels. Thus, the label shape did not match the anatomical structures in these areas.

Therefore, the purely conducting airways in the segmentation images were separated from the insufficiently captured gas exchange regions. To achieve this, a plane was drawn in ITK-SNAP v.3.6 perpendicular to the longitudinal axis through the airway at every location where a conducting airway (terminal bronchiole) transitioned into a respiratory airway, separating the structures. This transition was defined as the place where the surrounding airway epithelium changed from a thick, cuboidal to a thin, squamous shape [35]. Due to the fixation of the epithelium with osmium tetroxide and uranyl acetate, a change in contrast was observable at this location. As a second indicator for the transition, the increase in airway diameter and surface roughness of the 3D segmentation image at the transition into the gas exchange regions was used. Finally, an ITK filter was employed to remove all but the largest object in the segmentation image, thus reducing it to the conducting airway tree.

### Combination of *µ*CT and light microscopy

For the following histological analysis, the segmentations extracted from the *µ*CT images, containing the 3D context were combined with the high resolution LM tissue images. A detailed description of this workflow has been published by Grothausmann et al. [36].

The image combination was a three step process. First, the LM slices of each sampling section were registered to each other to create stacks of usually 24 slices. About 4% of the slices had to be excluded due to damages sustained in the cutting process. This first registration was performed to create a 3D LM volume with sufficient detail to allow registration between LM and *µ*CT images.

The segmentation images cannot directly be registered to the LM images, due to lack of common features. Therefore, a tissue-to-tissue registration between every one of the four lung *µ*CT volume images and each of the corresponding LM stacks was conducted. The adjustment parameters resulting from this registration could then be used on the segmentation images. This final step adapted the segmentations in a way that allows them to be overlaid on the corresponding LM images. Color coding of the segmentation allowed the introduction of specific markings into the LM images, e.g. IDs for measurement attribution or the clustering results, as shown in Fig. 2.

### Morphological feature acquisition

Measurements were collected per branch segment (section between two bifurcations, or one bifurcation and either the start of the segmentation image at the hilum or at its end at the end of the terminal bronchioles) in the LM images (three images per section). Segments were marked with an ID and color coded to facilitate data collection, as described by Labode et al. [15]. For the morphological analysis of airway lumen and wall dimensions, Fijis [37] line drawing tool was used to measure perpendicularly to the long axis of each airway cross section. Measurements were acquired for inner diameter (lumen) and outer diameter, measured from the outside of one airway wall to outside on the opposite side of the airway. The wall was defined as the mucosal and muscular tissue of the airway. Loose connective tissue surrounding the airway into its environment was excluded. The wall thickness was defined as follows:

1 $${\text{Wall}}\,{\text{thickness}} = \frac{{({\varnothing _{outer}} - {\varnothing _{inner}})}}{2}$$

If one segment was present in multiple consecutive images, its measurements were averaged.

In addition to these dimensional measurements, the airway epithelium was characterized following the principles of the LM study of rabbit airways conducted by Plopper et al. [38]. Cell counting and characterization were again conducted on a per ID basis. Measurements were performed in the 40x objective lens magnification scans of the center slice of the 24 slice LM sample sections. This was done using the software Zen Blue (Zeiss, Germany). For the lungs with 100 *µ*m stack distance, only every second stack center was analyzed, to make the results between lungs comparable. Along each airway segment, identified by an ID, a digital ruler with a length of 100 *µ*m was placed. Counting of cell nuclei for the categories ciliated cells, goblet cells, basal cells, club cells and other cells along this line was then performed. Additionally, a measurement of epithelial height and (if applicable) cilium length, each at this location was acquired, again using a digital ruler. To account for cilia positioned at an angle, the length measurement was conducted along the spine of the cilium, not in a vertical line from tip to cell membrane.

The researchers conducting the measurements and cell counts were blinded in regard to the group identity of the samples.

### Clustering

Different grouping methods were employed in this study to divide the airway tree into sub-compartments. The Bayesian information criterion (BIC) [39] in conjunction with a Gaussian mixture model (GMM) [40], calculated on the airway lumen and wall thickness, represents the central method of this study that is to be evaluated. These algorithms were used in their R [41] implementation in the package mclust [42]. Generations, orders and Strahler orders as branching-based grouping methods were also employed to provide a baseline of established method results for comparison. In short, generations count airway bifurcations from the trachea down to the periphery [16]. Orders count from the periphery inward and increase at each point were two or more branches meet [17]. Strahler orders count in the same way as orders, but do not increase at every intersection, instead they only increase when two or more segments of the same order meet [19]. These were determined using the Generation Analysis Toolkit [43].

To assess the grouping results provided by these methods, cluster quality metrics were employed. The Davies-Bouldin index [44], which calculates the average similarity between the clusters identified in a dataset, was utilized in its R implementation [45]. A well clustered dataset minimizes this value, i.e. produces groups that have little overlap. Additionally, the Dunn index [46] was employed, again by using an R package [47]. It lists the distance between the two closest clusters in a dataset and is thus not an overall metric, but instead presents only the ‘worst case’. For this metric, a large distance relates a good cluster separation.

### Statistical analysis

Comparisons of measuring results between NOX and HYX groups were performed using the Mann-Whitney U test [48] in its R implementation. The significance level was defined as 5% (*p* ≤ 0.05), effect strength calculations were performed as published by Rosnow and Rosenthal [49] and interpreted according to Cohen [50]. Means and standard deviations of the datasets were determined using the function ‘describeBy’ provided by the R package psych [51]. Plots were created using ggplot2 [52]. The measurements were analyzed as collected, with no outlier removal or other changes to the data.

## Results

### Clustering

**Fig. 1:**
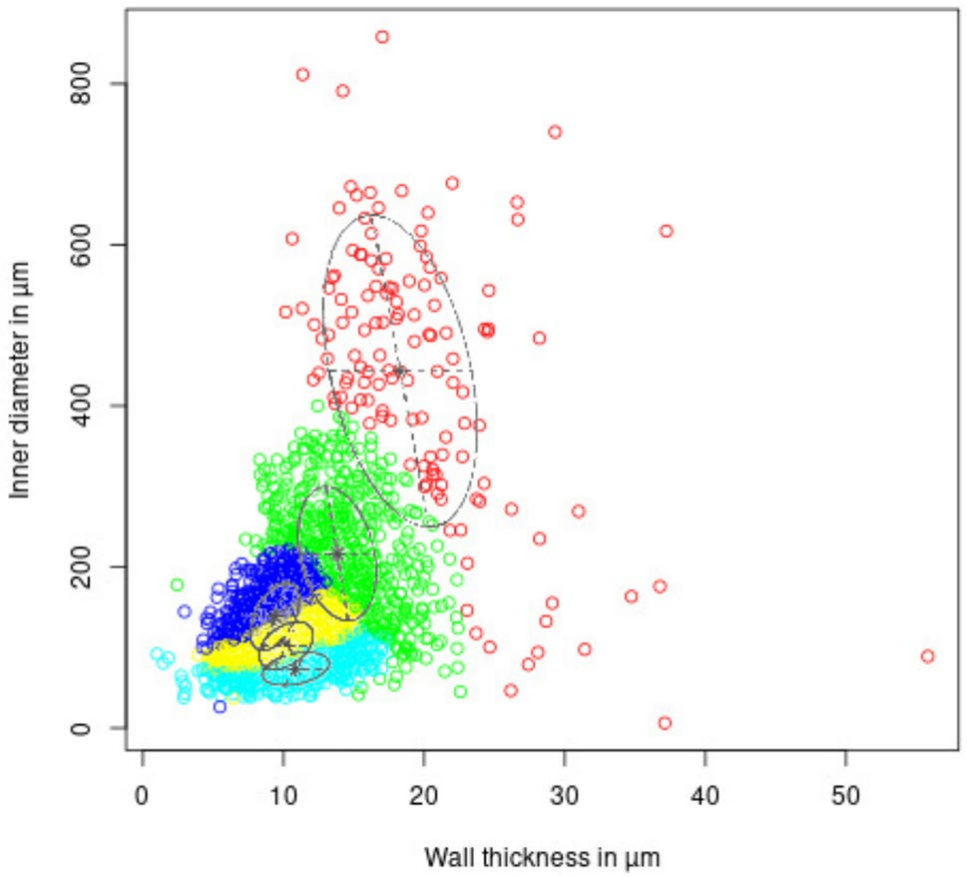
Cluster distribution within the morphological data of all four lungs. Clusters are marked by individual colors. Their centers and orientations of the underlying Gaussian distributions are marked with ellipses

The BIC delivered the highest score for five clusters in the dataset. The GMM further identified a cluster constellation of ellipsoidal distribution with variable volume, variable shape and variable distribution. Figure 1 shows the morphometric data of all vessel trees. The identified clusters are color coded, ellipses mark the identified Gaussian distribution centers and orientations.

Table 1 lists the results of the Davies-Bouldin index and the Dunn index, as well as the number of resulting clusters for each clustering method. While the GMM could be calculated over the complete morphological dataset of all four lung samples, the branching-based methods had to be applied to each tree structure individually. The results are therefore listed as mean values over all four lungs with their standard deviation.

**Table 1:** Davies-Bouldin and Dunn index results per grouping algorithm, with the GMM calculated over the data of all samples, while generations, orders and Strahler orders were applied to each sample. The number of resulting clusters per algorithm is listed as well

Algorithm	Davies-Bouldin index (SD)	Dunn index (SD)	Clusters (SD)
Generations	38.84 (9.74)	$1.26 \times {10^{ - 4}}$ ($3.79 \times {10^{ - 5}}$)	35.75 (2.13)
Orders	6.84 (1.76)	$2.44 \times {10^{ - 4}}$ ($2.48 \times {10^{ - 5}}$)	37.25 (2.56)
Strahler orders	3.28 (0.56)	$2.74 \times {10^{ - 4}}$ ($9.66 \times {10^{ - 5}}$)	6.75 (0.39)
GMM	2.09	$4.05 \times {10^{ - 4}}$	5

These results demonstrate that generations and orders are unable to divide the dataset into meaningful subgroups. While the Strahler order performs better, it is still outperformed by the GMM-based clustering scheme. It can thus be concluded, that the GMM-based approach performs best at dividing the dataset into morphologically homogeneous sub-compartments. It was therefore used as the basis for the following group-wise comparisons.

**Fig. 2:**
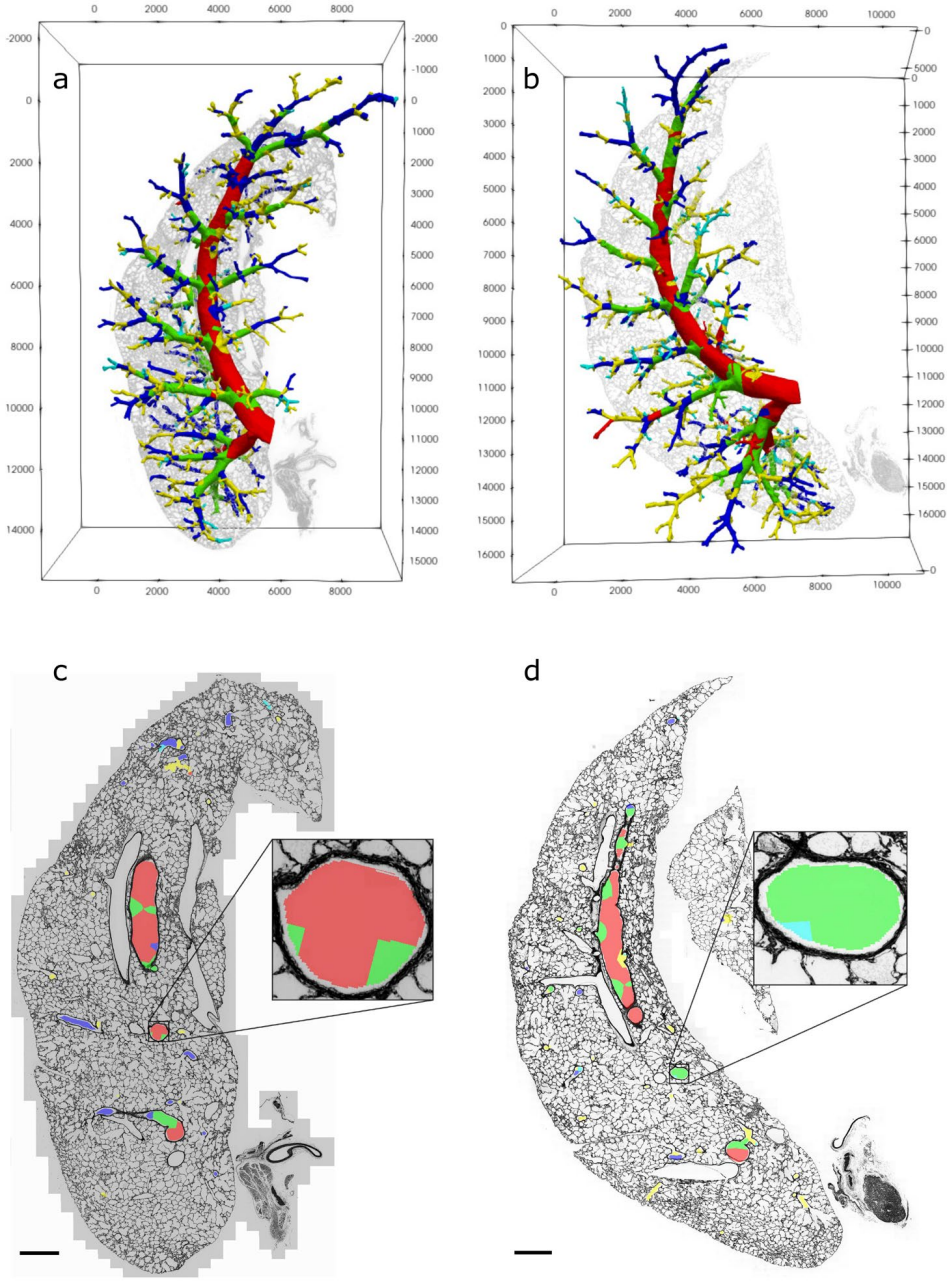
GMM clustering in the airways. **a** and **c** NOX 2, **b** and **d** HYX 2. Scale in **a** and **b** in *µ*m, scale bar **c** and **d** = 1 mm, insets are 8x magnified

Figure 2 displays the GMM clustering results mapped back into one airway tree per NOX and HYX group (Fig. 2a, b), as well as a 2D slice each with color coded airway lumina (Fig. 2c, d). This demonstrates comparability of results between animals and allows a characterization of the identified clusters (in combination with Fig. 1), listed here in descending order of lumen diameter:

- Red cluster (CL1): large, central airway of each lobe
- Green cluster (CL2): medium airways, distal of the large ones
- Blue cluster (CL3): small airways, larger diameter and thinner walls than the other small airways, distal of the medium airways
- Yellow (CL4): small airways, medium diameter and medium walls compared to other small airways, often distal of the blue ones, but sometimes directly distal of the medium airways
- Cyan (CL5): small airways, smallest diameter but highest spread in wall thickness of the small airways, small, most distal segments

### Airway lumen

**Fig. 3:**
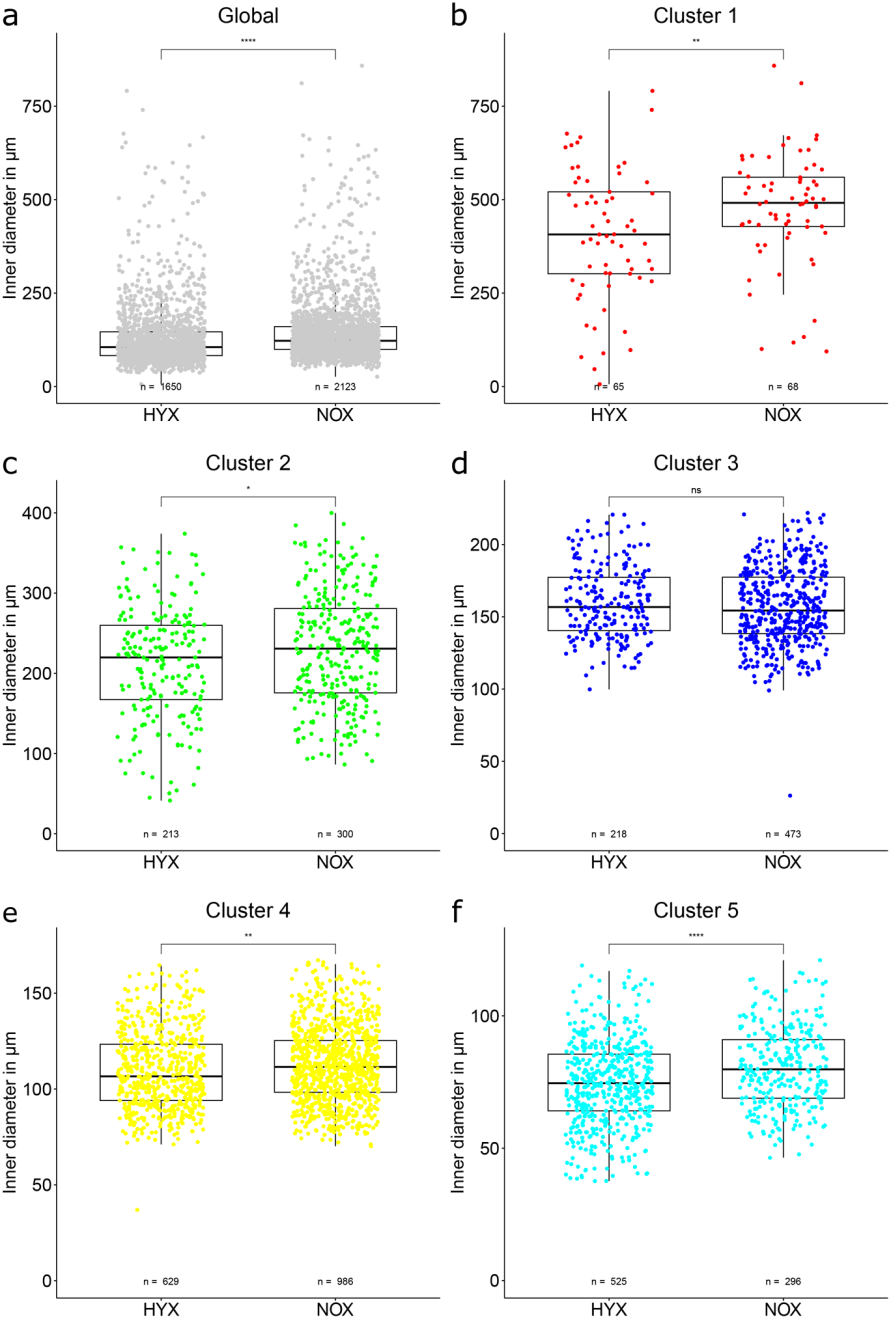
Lumen diameter comparison between HYX and NOX animal groups. Both global comparison and comparisons between clusters are displayed. Each data point represents one vessel segment. The box marks the range from the first quartile to the third quartile, the median is indicated by a horizontal line. The length of the whiskers is set to 1.5 times the interquartile range. Statistical differences between groups are indicated by: ns = *p* > 0.05, * = *p* ≤ 0.05, ** = *p* ≤ 0.01, *** = *p* ≤ 0.001, **** = *p* ≤ 0.0001

A global analysis, comparing all collected luminal diameters between treatment groups, shows the lumen diameter to be significantly smaller in the HYX group compared to the NOX group (see Fig. 3a). While the difference is highly significant, the effect strength of this deviation is categorized only as a weak effect. Comparing the lumen diameters within the airway clusters (see Fig. 3b–f), clusters 1, 2 and 5 show significant differences with a small effect strength. The difference in cluster 4 is significant, but the effect is to be categorized as no effect. In cluster 3 there is no significant difference between groups. All significant differences within the clusters mirror the global pattern of a smaller median value in the HYX group compared to the NOX group.

### Airway wall

**Fig. 4:**
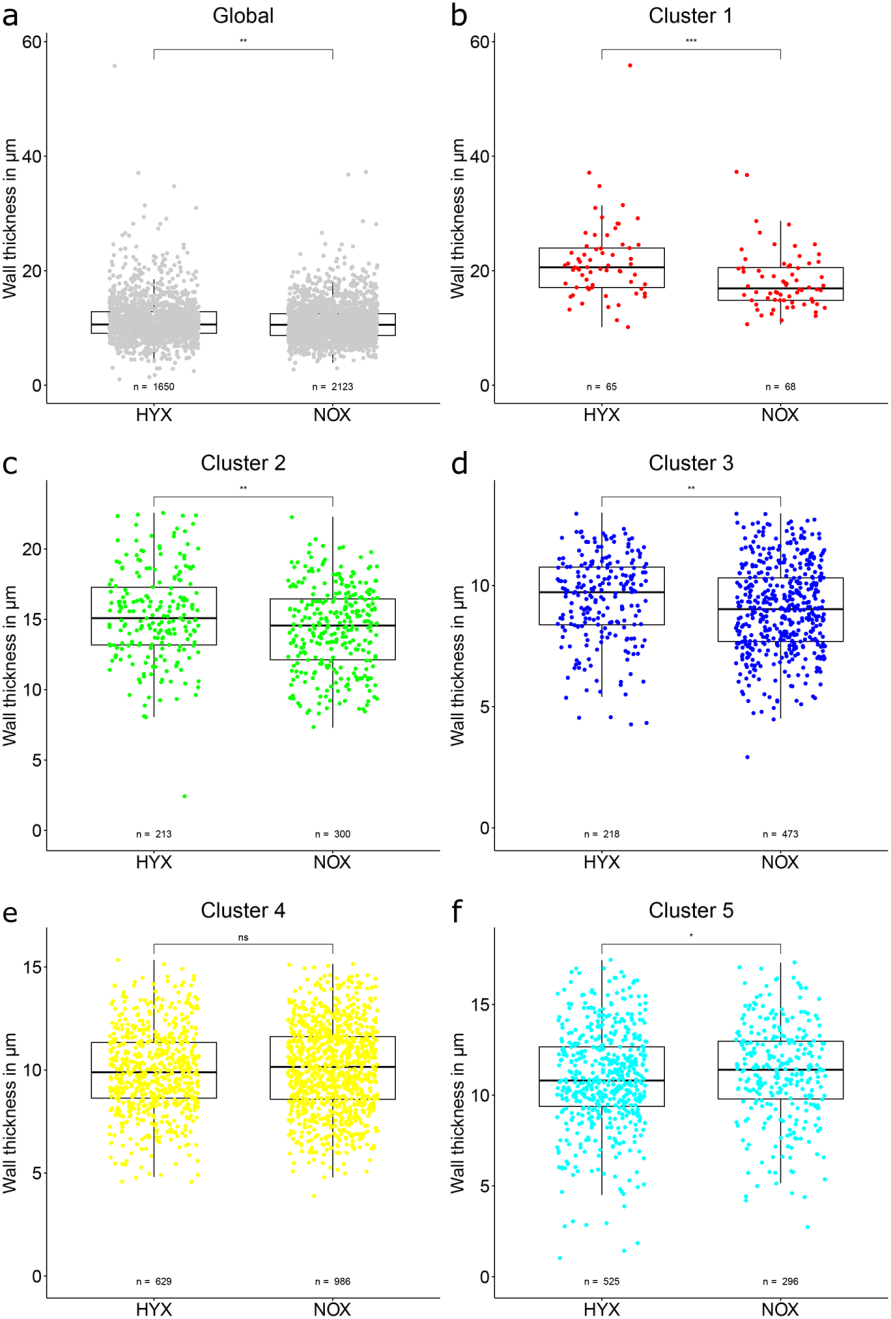
Comparison of airway wall dimensions between NOX and HYX animal groups. Both global comparison and comparisons between clusters are displayed. Each data point represents one vessel segment. The box marks the range from the first quartile to the third quartile, the median is indicated by a horizontal line. The length of the whiskers is set to 1.5 times the interquartile range. Statistical differences between groups are indicated by: ns=*p* > 0.05, * = *p* ≤ 0.05, ** = *p* ≤ 0.01, *** = *p* ≤ 0.001, **** = *p* ≤ 0.0001

A global analysis shows a significant difference in wall thickness between NOX and HYX animals (see Fig. 4a). While significant, the absolute difference in numbers is slim with a median value of 10.61 *µ*m for the HYX group and 10.55 *µ*m for the NOX group. The effect strength is to be interpreted as no effect.

The results are more conclusive when looking at the individual clusters (Fig.4b–f). In cluster 1, the higher wall thickness in the HYX group compared to the NOX group reaches a medium effect strength. The same, but less pronounced situation is observable in clusters 2 and 3, where a weak effect is present. There is no significant difference between groups in cluster 4. In cluster 5, the ratios are flipped with a higher median wall thickness in the NOX group, but the effect strength is categorized as no effect.

### Airway epithelium

**Fig. 5:**
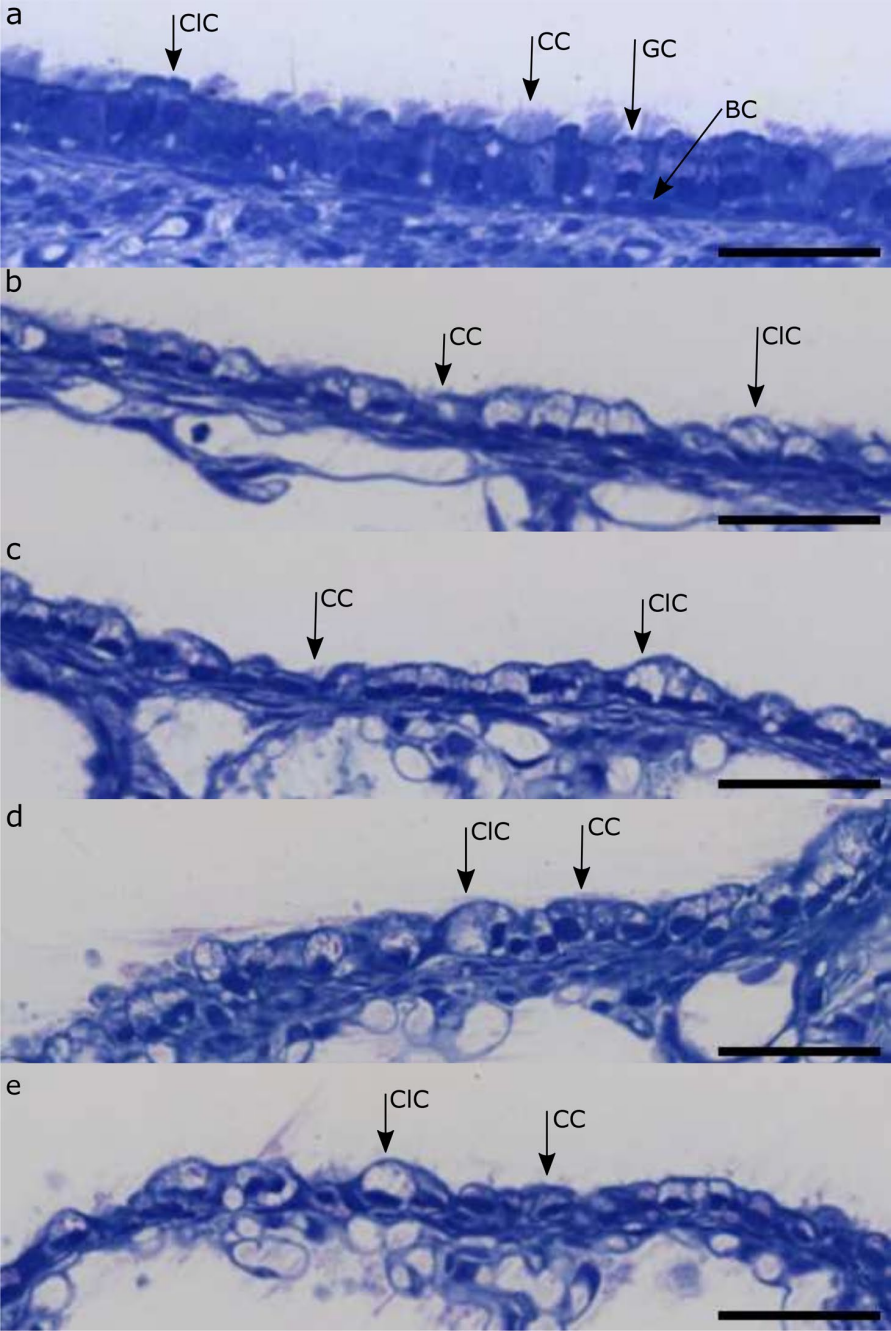
Representative sections of airway epithelium for each cluster. a: Cluster 1, b: Cluster 2, c: Cluster 3, d: Cluster 4, e: Cluster 5. Sample NOX 2, scale bar = 40 *µ*m. CC = Ciliated cell, ClC = Club cell, GC = Goblet cell, BC = Basal cell

Results of the dimensional and density analysis of the airway epithelium are listed in Table 2. In total, measurements for 685 segments for the HYX group and 799 locations for the NOX group were collected. Figure 5 shows exemplary light microscopic images of different sections of the airway epithelium of one animal.

**Table 2:** Airway epithelium analysis. Statistical differences between groups are indicated by: ns=*p* > 0.05, * = *p* ≤ 0.05, ** = *p* ≤ 0.01, *** = *p* ≤ 0.001, **** = *p* ≤ 0.0001

Parameter	Cluster	Mean HYX (SD)	Mean NOX (SD)	*p*-value	Effect strength
Epithelium height (µm)	Global	10.06 (2.57)	7.79 (1.44)	****	Medium
	CL1	11.88 (2.45)	9.57 (1.58)	****	Large
	CL2	10.95 (2.84)	8.36 (1.39)	****	Medium
	CL3	8.26 (1.75)	7.47 (1.23)	***	Small
	CL4	9.26 (2.14)	7.44 (1.29)	****	Medium
	CL5	11.13 (2.31)	7.75 (1.24)	****	Large
Cilium length (µm)	Global	5.10 (1.46)	5.79 (1.36)	****	Small
	CL1	6.44 (0.98)	7.22 (1.31)	**	Small
	CL2	5.70 (1.35)	6.17 (1.33)	*	Small
	CL3	5.13 (1.02)	5.56 (1.15)	**	Small
	CL4	4.77 (1.30)	5.24 (1.15)	**	Small
	CL5	4.33 (1.59)	5.34 (1.03)	**	Small
Density (nuclei/mm)	Global	103.62 (22.55)	115.01 (20.11)	****	Small
	CL1	112.40 (22.02)	136.20 (17.91)	****	Large
	CL2	107.49 (23.51)	116.77 (19.22)	***	Small
	CL3	110,00 (18.97)	115,83 (19.72)	ns	-
	CL4	103.49 (21.50)	111.30 (19.18)	****	Small
	CL5	94.91 (22.73)	108.40 (17.42)	****	Small

The airway epithelium height shows a significant difference between groups with the HYX group showing a larger mean epithelial height in a global comparison. The effect strength is medium. This pattern is clearly visible in all clusters with high significance and several medium and large effect strengths. Only in cluster 3 the difference is less pronounced with a small effect strength.

The cilium length shows a significant global difference between groups, with the NOX group exhibiting a higher mean cilium length than the HYX group. The effect strength is small. This difference is observable and significant in all clusters with an universally small effect strength.

The density of cellular profiles (expressed as nuclei per mm) is significantly higher in the NOX group compared to the HYX group in a global comparison. Here, the effect strength is small. A cluster-wise comparison shows a more differentiated situation. In the large airways (cluster 1), a significant difference like in the global comparison is present, but with a large effect strength. Clusters 2, 4, 5 in contrast still show a significant difference of this kind, but only a small effect strength. Cluster 3 is the only cluster with no significant difference between groups.

The cell composition of the airway epithelium is listed in Table 3, expressed as percentage of observed nuclei for each cell type.

**Table 3:** Airway epithelium composition. Statistical differences between groups are indicated by: ns = *p* > 0.05, * = *p* ≤ 0.05, ** = *p* ≤ 0.01, *** = *p* ≤ 0.001, **** = *p* ≤ 0.0001

Parameter	Cluster	Mean HYX (SD)	Mean NOX (SD)	*p*-value	Effect strength
Ciliated cells (%)	Global	47.89 (12.06)	46.12 (10.26)	**	None
	CL1	53.27 (14.26)	53.99 (10.75)	ns	-
	CL2	51.16 (10.26)	47.85 (9.93)	*	Small
	CL3	46.50 (8.58)	45.71 (9.84)	ns	-
	CL4	45.35 (11.49)	44.91 (11.49)	ns	-
	CL5	48.57 (13.96)	42.03 (10.87)	**	Small
Club cells (%)	Global	48.41 (13.11)	51.74 (10.71)	****	Small
	CL1	39.94 (15.19)	41.12 (13.48)	ns	-
	CL2	44.70 (12.72)	49.23 (10.38)	*	Small
	CL3	51.99 (9.59)	52.45 (9.70)	ns	-
	CL4	51.43 (12.15)	53.50 (9.75)	*	None
	CL5	46.96 (13.94)	56.46 (10.07)	****	Medium
Basal cells (%)	Global	3.01 (7.07)	1.80 (3.91)	**	None
	CL1	5.33 (7.28)	3.64 (5.31)	ns	-
	CL2	3.58 (7.74)	2.45 (4.30)	ns	-
	CL3	1.42 (4.01)	1.74 (3.73)	ns	-
	CL4	2.81 (7.93)	1.32 (3.51)	**	Small
	CL5	3.16 (6.31)	1.21 (3.44)	*	Small
Goblet cells (%)	Global	0.09 (0.87)	0.05 (0.60)	ns	-
	CL1	0.70 (2.22)	0.60 (1.86)	ns	-
	CL2	0.25 (1.48)	0.06 (0.72)	ns	-
	CL3	0.00 (0.00)	0.00 (0.00)	-	-
	CL4	0.00 (0.00)	0.00 (0.00)	-	-
	CL5	0.00 (0.00)	0.00 (0.00)	-	-
Other cells (%)	Global	0.59 (2.94)	0.28 (1.74)	*	None
	CL1	0.76 (4.82)	0.64 (3.15)	ns	-
	CL2	0.30 (1.67)	0.41 (2.16)	ns	-
	CL3	0.09 (0.85)	0.11 (1.09)	ns	-
	CL4	0.40 (2.01)	0.27 (1.55)	ns	-
	CL5	1.31 (4.42)	0.30 (1.47)	ns	-

In a global comparison, there is a significant difference in ciliated cell percentage present between groups, with the HYX group showing a higher mean value. While significant, the difference is numerically small and the effect strength is categorized as no effect. Local differences of significance are only present in clusters 2 and 5 with a small effect strength each time.

The club cell percentage shows a significant difference between groups in a global comparison. The NOX group exhibits a higher mean value. The effect strength is small. No local significance is present in clusters 1 and 3. A significant difference but no effect can be observed in in cluster 4. Significant differences with relevant effect strength are evident in cluster 2 (small effect) and cluster 5 (medium effect).

For the basal cells, a significant difference between clusters can be identified in a global comparison, with the HYX group showing a higher mean percentage than the NOX group. The effect strength is classified as no effect. Significant local differences are only found in clusters 4 and 5 with a small effect strength for both.

Goblet cells could only be identified in the clusters containing larger airways (clusters 1 and 2). The absolute numbers are quite low. No global or local difference of significance was identified.

The percentages of other cells, e.g. neuroendocrine cells, brush cells or cells that could not be identified as one of the other listed categories with confidence, show a global difference. It is significant but only by a slim margin with a p-value of 0.045. The HYX group presents a higher mean percentage compared to the NOX group, albeit with very low effect strength. Local analyses reveal no significant differences.

## Discussion

### Airway morphology analysis

The airway lumen analysis shows a globally smaller lumen in the HYX animals compared to the NOX animals. This is in line with the results of e.g. Mühlfeld et al. [25], who reported a significant reduction of ductal airspace volume with hyperoxia treatment of the premature rabbit. The model therefore mirrors the situation in humans, where a smaller mean bronchiolar diameter in children with BPD is found [53]. The cluster-based analysis further shows that the effect is mostly found in the two clusters containing larger diameter airways (clusters 1 and 2). In the three small ones, no significant difference (cluster 3), no effect (cluster 4) or only a small effect (cluster 5) could be identified. While the number of alveoli often catches up with that of healthy individuals later in life, the airway lumen reduction observed in BPD can persists into late childhood and even adulthood [54]. Detailed knowledge about the location of this dysanapsis is therefore important for targeted mitigation strategies.

The airway wall analysis returns similar results to the more focused epithelial height analysis (see following section). The wall thickness of the HYX group is significantly higher both globally, as well as in clusters 1, 2 and 3. The cluster-based analysis is able to pinpoint the highest expression of this change to the large airways (cluster 1) with a medium effect strength. In cluster 4 there is no significant difference and only in cluster 5 there is a higher wall thickness in the NOX group, but only with negligible effect strength. These results plausibly reflect the sum of multiple known remodeling processes in human BPD or respective models. Publications list for example increased epithelium height in the airways [26, 55], variable airway smooth muscle hyperplasia [56] and also a thickened subepithelial collagen layer (approximately twofold) after hyperoxia exposure [57].

### Airway epithelium analysis

Looking at the results of the airway epithelium analysis in the order they are listed above, the first parameter (see Table 2) is the epithelium height. An increase of this value in the HYX animals compared to the NOX animals could be identified. Epithelial thickening has been reported for BPD in humans [58] and is also in line with the results of Gie et al. [26]. In that study of 32 animals (13 NOX and 19 HYX rabbit pups, prematurity and age identical to the present study), epithelial height was measured in ten random airways per animal. The cluster-based analysis conducted in the current study adds to that knowledge and shows that the increase is a global pattern that could be identified in all sections of the conducting airways.

The extensive data published by Gie et al. [26] shall also serve as a baseline to discuss the inter-animal variance in the present study. For the NOX animals, that study reports mean epithelial heights per animal ranging from 6.75 *µ*m to 8.25 *µ*m, with an average of 7.50 *µ*m. The two NOX animals investigated in the current study present mean values of 7.92 *µ*m (NOX 1) and 7.67 *µ*m (NOX 2) respectively. They are thus well within the larger studies range and can be judged as an appropriate representation. The HYX rabbits in the reference publication show mean epithelial heights ranging from about 8.50 *µ*m to 10.75 *µ*m with an average of 9.50 *µ*m. The animals of the present study show means of 11.64 *µ*m (HYX 1) and 8.32 *µ*m (HYX 2). They are thus located at each end of the spectrum published previously. They present similarly in other aspects as well, with the NOX animals showing similar values to one another, while the HYX animals exhibit more inter-animal differences. There is a general tendency of HYX 1 to express more intense disease-associated changes (e.g. a smaller airway lumen) than HYX 2. Overall, they are nevertheless deemed to be a reasonable representation of the changes associated with prematurity and hyperoxia treatment.

The next parameter surveyed, the cilium length, was found to be significantly shorter in the HYX animals compared to the NOX group. This difference was present in all sub-compartments of the lungs with an equal small but measurable effect strength. Shortened cilia have previously been reported in hyperoxic mice and cultured human tracheobronchial epithelial cells exposed to hyperoxia [59]. Ciliary abnormalities were also reported in a limited study on airways of premature infants with BPD [60]. The results presented here suggest that this phenomenon is present in premature, hyperoxia-treated rabbits as well. Confirmation should be acquired in a another, possibly retrospective study with a representative animal group size. Whether the small effect size measured is still large enough to significantly impact mucociliary clearance and facilitate disease progression will also have to be investigated further.

For the density of cell nuclei, there is a significant, albeit small global difference with the NOX group having a higher number of nuclei per mm than the HYX group. This might warrant the hypothesis that in the HYX animals there is not only a cell height increase, but a cell hypertrophy in all directions present. The cluster-based analysis further shows that the effect is most prominent in the cluster containing the large airways (cluster 1), while in the other clusters there is only a small effect (clusters 2, 4 and 5), or there is no significant difference between groups at all (cluster 3).

As the analysis of the airway epithelium in this study was based on the approach by Plopper et al. [38], the results of the current study will now be compared to the published data.

While the methodical approach of the original study was mostly replicated, there are differences that limit the comparison. The animals used in the current study were three days premature and one week old when the organs were extracted. The original study on the other hand used animals that were 8 to 15 weeks old. Another difference lies in the sampled structures. The original publication analyzed generations 0 to 7 of the airways, i.e. from the trachea down into the larger airways of both lungs. The present study on the other hand was limited to the left lung and the samples did not include the trachea and only the most distal section of the main bronchus, but all conducting airways were analyzed. The last difference between studies concerns the airway classification scheme. While originally generations were used, the current work uses a GMM clustering scheme for the reasons discussed above. To achieve a meaningful comparison, cluster 1 (largest vessels) of the NOX group will now be compared with the range of results for generation 2 to 7 published in the original study. Generations 0 and 1 are excluded, as these were not or only to a small extent present in the organ samples of the current study. For the interpretation it is also important to take into consideration, that cluster 1 includes more than seven generations and thus the mean values listed will be influenced by these more distal segments.

The epithelial height assessed in the original study ranges from 9.00 *µ*m to 16.00 *µ*m. For cluster 1 NOX, a mean value of 9.57 *µ*m was determined. This is at the lower end of the published range, which seems reasonable, considering the younger age and the more distal airway sections included in this cluster. As a general rule, the original study also states, that the epithelial height reduces to about half of that of the trachea in generation four and stays constant thereafter, which matches relatively similar heights measured in clusters 3 to 5 NOX. The cell density values are listed from 114.40 nuclei/mm to 180.50 nuclei/mm. Cluster 1 NOX again falls within this range with 136.20 nuclei/mm.

Looking at the cell population, the ciliated cells present between 39.60% and 53.60%. Cluster 1 NOX is outside of that range by a slim margin with a value of 53.99%. Club cell percentages are given as 24.70% to 47.30% with values steadily increasing with higher generations. Cluster 1 NOX is within that range with a value of 41.12%. The basal cells account for 0% to 26.5%, with their numbers gradually decreasing over the listed generations, reaching 0 in generation 7. Cluster 1 NOX is at the lower end of that range with 3.64%. Goblet cells were only found in generations 0 to 2. As the first two generations are excluded here, the only non-zero percentage is given for generation 2 with 0.7%. For cluster 1 NOX, the value is listed as 0.6%, so it is higher than the average of the range in the comparison study.

The ‘other cells’ category is the only one with a large deviation from the reference data by about one order of magnitude (3.9% - 7.5% originally reported in contrast to 0.64% in cluster 1 NOX of the current study). For the interpretation of this data it needs to be kept in mind that the other cell category is a ‘catch all’ category. It not only contains cell types such as brush cells or neuroendocrine cells, but also all cells, that were unidentifiable due to e.g. local cutting artifacts. Thus, a difference here might reflect a studies higher instance of tissue damaged in the preparation, differences in image quality or observer confidence opposed to actual variations in cell type proportions. The lower count in this category accounts for the higher percentages in the other categories in the results published here, mainly in the ciliated and club cell categories.

Moving on from the comparison between the NOX group and previously published data for verification purposes, the next step is the comparison between NOX and HYX groups within the current study. Looking at the group-wise comparisons (see Table 3), there are some significant differences in percentages present, but the effect strength generally ranges between small and none, with one outlier in the club cell percentage in cluster 5. So there seem to be no obvious signs of e.g. a major hyper- or hypoplasia of a certain cell type being present. Whether the differences identified in the current study are a small but constant sign of BPD or if they are just a symptom of inter-animal variability, would need to be investigated in a study with larger group sizes.

Another topic worth discussing is the fact, that several studies on human lungs report abnormalities in goblet cells in BPD [60] and prematurity [61]. These could not be verified in the current study due to very low cell numbers of this type in both groups. This seems to be rabbit-specific, as the study by Plopper et al. [38] on a larger sample size of healthy rabbit lungs was also only able to identify goblet cells in airway generations 0 to 2, ranging from 1.3% of the cell population in the trachea to 0.7% in the lobar bronchi and being absent thereafter. This is a major disparity to the human lung, where goblet cells are found throughout all conducting airways [62]. This circumstance thus seems to be a limitation of the rabbit model of BPD.

### General workflow approach, performance and limitations

Before going into its finer details, a discussion of the general workflow approach is in order. The underlying principle of the method is the grouping of airway segments from different treatment groups on a ‘macroscopic scale’ by overall similarities to then perform comparisons within these resulting clusters on a ‘microscopic scale’ to find deviations between the treatment groups. The major precondition for this method to work as demonstrated in the current study is that the lungs of the different treatment groups still share major morphological properties. This will not be the case for all pathologies. One can imagine different scenarios, where an adaption of the workflow would be necessary. The first problem that comes to mind would be branches in e.g. the experimental group being changed in a way that they convincingly mirror the morphological properties of a sub-compartment of unequal position of the control group. One might think of a hyperinflation of the airways in the second cluster that causes them to group with the first cluster. Such a change is less likely than the affected structures presenting different from the unaffected ones, as discussed below. If present, percentage-wise shifts in cluster attribution would be noticeable, as well as differences in 3D renderings like the one shown in Fig. 2a and b. Another problem that might be encountered would be that major morphological differences between treatment groups exist and equal sub-compartments between these groups cannot be identified. There is a mutual solution for both of these situations. If the branching pattern is still comparable between samples, the use of a branching based grouping scheme, such as the Strahler order would be encouraged. If the differences are very pronounced however, the purpose of a sub-compartment-wise approach should be questioned altogether. A more expected situation would be a change predominantly in one property. E.g. a large increase in wall thickness in the experimental group. In a plot like the one shown in Fig. 1 this would cause a shift to the right in the data points of the affected group. Should the effect be severe enough it would lead to the formation of additional clusters. In this situation, one could find clusters predominantly populated by one treatment group while other clusters would be mostly populated by the other group. In this case, the cluster analysis should be conducted individually for each treatment group. On this basis, a comparison could be performed. Again, should the differences between groups be too severe, the general applicability of this approach should be questioned.

In the current study, this workflow was able to provide morphological information, combined from undisturbed 3D *µ*CT volume images, as well as high resolution LM slice images. It was also able to divide the airways into meaningful homogeneous compartments, providing better insight into the distribution of airway changes in a two group comparison study.

Nevertheless, the workflow showed limitations. These are mainly in the area of scalability. The steps of image segmentation, airway trimming and measurement acquisition were done with a high degree of manual interaction. While feasible for a study of the presented size, a study with e.g. n = 10 animals per treatment group would currently require an unreasonably high amount of manual work. For future studies, a higher degree of task automation should therefore be ensured.

To achieve this goal, the data gathered in this study can serve as an important baseline to compare against. The segmentation that was conducted in a semi automated fashion in the current study can be replaced with a deep learning approach for lung image segmentation [63, 64]. A recent study by Schmidt et al. published a workflow for the automated segmentation of capillaries in the rabbit model of BPD, trained on analogous segmentations to the ones created in the current study [65].

The dimensional measurements of lumen diameters per ID can be automatically calculated using Fijis local thickness algorithm [66]. The same algorithm could be employed for wall thickness measurements. For this, the airway wall would need to be segmented as well, which could be achieved with little effort by threshholding and binarizing the *µ*CT images. The step of associating IDs of the airway lumen segmentation with the bordering wall is however a more complex task, but can be assumed to be solvable using a nearest neighbor algorithm [67].

Lastly, the number of necessary counting events for representative cell assessment could be investigated by simulating other counting patterns (e.g. increasing step size between images) and thus identify the effective number of images to analyze for representative results. This manual cell investigation might also be removed in its entirety by using an automated cell density analysis, as developed by Jang et al. [68].

Also, the counting process in the cellular analysis did not use a spatially unbiased method. This was due to the choice to replicate the procedure used by Plopper et al. [38], in an effort to produce results comparable to this large scale study for verification purposes. Future studies should instead employ unbiased stereological counting methods [69]. These are compatible with the clustering approach employed here as discussed by Mühlfeld et al. [70]. This would ensure that the cells sampled are representative for the corresponding cell population.

Another aspect was the preparation of the histological sections that limited the identification of different airway epithelium cells. The present study is a secondary analysis of material that was originally created for the analysis of the pulmonary vasculature [30]. There, the main focus was the registration of *µ*CT and LM images. Thus, a toluidine blue staining procedure was chosen, resulting in a single color stain with an intensity proportional to the density of the structures. The dense basal bodies in ciliated cells were therefore visible as a dark apical line (see Fig. 5a), aiding identification. Other cell types with less prominent morphological characteristics however proved more challenging to identify. Marking techniques such as periodic acid-Schiff (PAS) staining for the identification of mucus filled goblet cells [71] or immunohistochemistry staining on club cell protein for the identification of club cells [72] would have been useful additions, but could not be employed retroactively on the already embedded tissue. In future studies, these techniques might be included from the start to facilitate the epithelial analysis.

### Clinical status quo and possible method applications

Currently, the diagnosis of BPD is based on the need for supplemental oxygen [73], without the integration of a medical imaging protocol. Thus, the site and the extent of pathological changes in the patients lung are usually unknown. This hampers targeted individualized therapy.

Chest radiography can be employed as an imaging method to assess the severity of BPD, but CT examinations show higher correlations with clinical outcomes [27, 28]. Both of these methods rely on radiologist interpretation and are thus susceptible to inter and intra observer variability in the resulting scores. CT scans of the airways have proved to correlate changes in airway lumen and wall dimensions with the intensity of mechanical ventilation and supplemental oxygen use in patients [29]. The cluster based method demonstrated in the current study, especially with additional automation, could improve the precision of these methods and exclude human interpretation, providing objective scores. The severity assessed in this fashion could serve as a basis for identifying critical cases that require close monitoring.

Pediatric CT examinations come with severe risks. Estimations put the lifetime cancer mortality risk due to the radiation dosage received in a pediatric CT about one order of magnitude higher than that for adults [74]. It is thus not a suitable tool for routine examinations, but may be used upon indication to support intervention decisions, predict rehospitalization risks and the need for extended oxygen or medication therapy. Also, future developments might reduce radiation dose and thus lower the risks associated with pediatric CT scans. Alternatives, such as ultrasound [75] and magnetic resonance imaging (MRI) [76] are able to circumvent the risks of radiation exposure, but only provide limited image resolution.

The clinical relevance of BPD is not limited to neonates. Premature birth in general and specifically BPD have lifelong implications. Medical imaging is important for tracking disease progression through childhood and well into adulthood. A study of 16–23 year old BPD survivors showed structural abnormalities in the 92% of the patients lungs. This included bronchial wall thickening. A higher extent of abnormalities was closely related with decreased lung functions and respiratory hospitalizations [77].

A systematic lung CT assessment can show changes associated with BPD and quantify severity and probable outcomes. A lack of well-defined anatomical subtypes of BPD currently prohibits targeted individualized therapy. This study lays the groundwork to better define the site of disease and identify subgroups of BPD allowing for the investigation of targeted therapy.

## Conclusions

The global results of the small scale proof of principle study presented here are in line with those of previous studies containing larger sample sizes. On this data it could be demonstrated that the GMM-based approach is usable for dividing a monopodial airway tree into meaningful sub-compartments. It outperformed other established methods for this task.

The method further proved to be successful in uncovering differences between the two experimental groups in a higher resolution than possible with an unfocused, global approach. Its future use should be in a full-size study with a representative sample size to gather information about the manifestation of the studied disease in the different lung sub-compartments.

## Data Availability

The datasets used and/or analysed during the current study are available from the corresponding author on reasonable request.

## Abbreviations

*µCT*: Micro computed tomography
BIC: Bayesian information criterion
BPD: Bronchopulmonary dysplasia
CT: Computed tomography
GMM: Gaussian mixture model
HYX: Hyperoxic
ITK: Insight Toolkit
LM: Light microscopy
MRI: Magnetic resonance imaging
NOX: Normoxic
PAS: Periodic acid-Schiff
